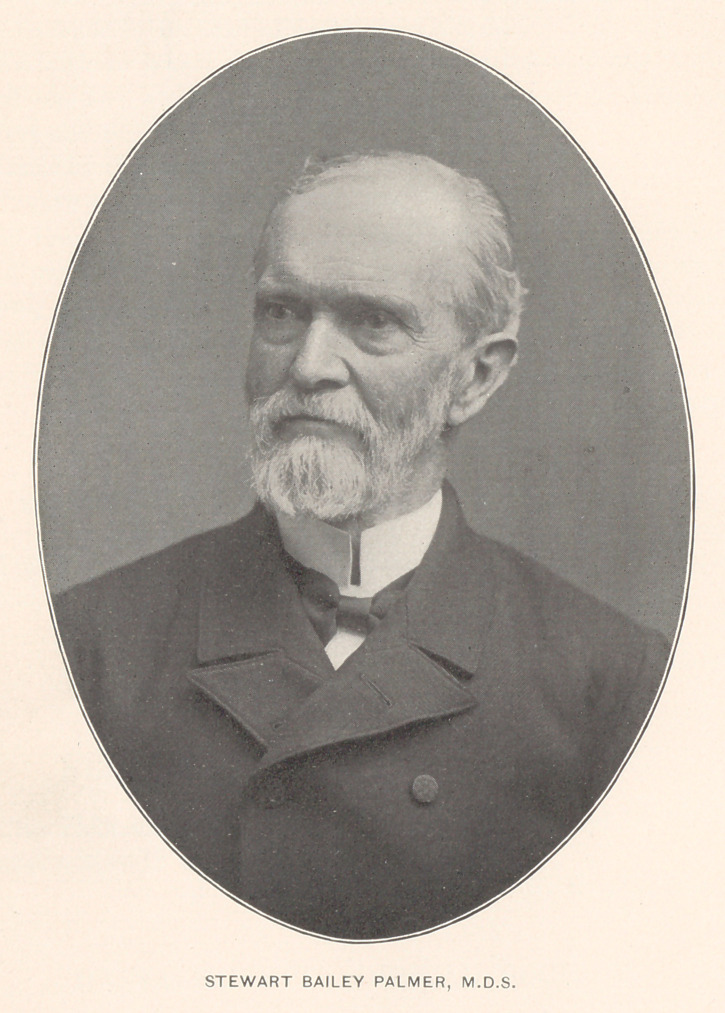# Stewart Bailey Palmer, M.D.S.

**Published:** 1903-05

**Authors:** 


STEWART BAILEY PALMER, M.D.S.

### Obituary.

### STEWART BAILEY PALMER, M.D.S.

A NOBLE man, who in practising our profession bestowed honor on it, has passed away from our midst.

Stewart Bailey Palmer, M.D.S., died at his home in Syracuse, N. Y., on March 30, 1903, aged eighty years, six months, and thirty days.

He was one of the pioneers of dentistry in the section of the country where he lived and where he has had a long professional life marked by the constant endeavor to keep his work up to the highest standard possible of professional excellence. That he succeeded in this is evidenced by the high esteem in which he was held by all those on whom he displayed his manipulative skill.

Dr. Palmer was one of those men whom circumstances and environment cannot suppress. Born and brought up on a farm, far away from any village, he received but scanty training from the country school of the district where he resided, as his duties at home prevented him from attending the school regularly, but the limited education acquired there did not satisfy his thirst for knowledge, for he was a born investigator, and when quite a boy a copy of Comstock's Philosophy, which was placed in his hands at school, fired his ambition to know all about the various subjects of which it treated, and thenceforth every moment he could spare from his regular duties was devoted to study and the acquisition of knowledge. In this way he qualified for a course at the Cortland Academy, a high-grade school, which he entered and took one term of instruction, which was all he could afford at that time. He finished this course in 1846, being then twenty-four years of age, but he was still unsatisfied, and he longed to be able to study and experiment along the lines of the knowledge he had acquired there. To do this he must have tools and material, but he had no means with which to get them. Shortly after he left school he was offered the position of teacher of the district school at Tully for the next year,—1847. This he gladly accepted. Here was an opportunity to get tools and whatever he wanted to pursue the studies that would give him the knowledge he longed for. With the means thus acquired, and amid many difficulties, he went to work and succeeded in constructing working models of most of the implements and machines described and pictured in Comstock's Philosophy, which was the leading text-book used in the schools at that time. Among those he constructed were a working model of a steam-engine, an electric machine, a galvanic battery, etc. Here he was storing up useful practical knowledge and gaining manipulative skill in departments of science, which were soon to be put to a practical test that was to determine the destiny of his life. His teeth were of poor quality, and many of them were destroyed by disease early in life.

During the year 1847, while he was teaching school, he had nine of them extracted, but he could not then afford to pay for an artificial set of teeth. At that time the plates on which artificial teeth were mounted were made of either gold or silver. Although up to this time he had never been inside of a dental laboratory, or examined closely an artificial denture, the loss of his teeth made him very uncomfortable, and he had no money to have an artificial set made. As he became skilful in the use of tools, and the discomfort of his mouth annoying him, he often wondered if he could not make something that would relieve his discomfort. While he was in this condition he happened to go into a drug-store in Syracuse, and saw lying in a case on the counter a book with a set of artificial teeth delineated on the cover. He asked the druggist to let him see it, which he did, and upon examining it he found it was a treatise on dentistry, with illustrations, describing the mode of constructing artificial dentures. He asked if the book was for sale, and was told that it was and the price was five dollars. He did not have that much money with him, but he went to a friend of his father's in that town and borrowed three dollars from him, and went to the store and bought the book. He studied the contents until he thought he had acquired the knowledge he wanted, and determined to try and make a set for his mouth. He hammered out a plate from a silver dollar on an anvil in a blacksmith's shop, and thus equipped, commenced to make a set of artificial teeth to supply the deficiencies in his own mouth. He succeeded in doing this, and so well that he was solicited to make several artificial dentures for other people. In all the cases of this kind that he undertook his success was so complete that he determined to adopt dentistry as the occupation of his life. And thus he embarked in a calling in which he was destined to become celebrated.

Having decided upon this course, he devoted all of his indomitable energy to acquire a thorough knowledge of all the best methods as practised by the eminent men of his chosen calling in those days. To gain this information so much desired, he sought and obtained interviews with all the distinguished dentists he could reach, and it is needless to write that he succeeded in obtaining the desired information.

In the following year, 1848, he became associated in dental practice with Dr. John L. Allen, at Fabius, which association continued until 1850, when he started in practice by himself, in Lafayette. In the same year he married Miss Elizabeth Jane Savery, now deceased.

In 1851 he moved to Tully, and remained in practice there until 1866, when he removed to Syracuse and entered into partnership with Dr. Amos Wescott, with whom he remained until 1868, when they separated, and he opened an office for himself; and from that time until he retired, about two months before his death, a period of thirty-five years, he continued to practise his profession in the city of Syracuse, making altogether a continuous practice of fifty-five years.

He has stated that from the moment he entered the dental profession his education has been a continuous advance in his profession throughout the whole of this long term. He said he was constantly stimulated by a desire for thorough knowledge on his part, and also stimulated and aided by dental literature and attendance upon dental meetings. Early in his practice he became a member of the American Dental Association, joining the latter in 1864 and always retaining his membership. In 1876 he was elected a member of the New York Odontological Society. He assisted in the organization of the Dental Society of the State of New York in 1853. In 1868 he received from the State Society (New York) the degree of M.D.S., Master of Dental Surgery. He was also an officer of the Fifth District Dental Society of the State of New York and a member of the Syracuse Dental Society. In 1872 he was elected a member of the Board of Censors of the Dental Society of the State of New York, and continued a member of that board until 1895, when the Legislature of New York gave the power of granting licenses to practise dentistry to the Regents of the University, and created a Board of Examiners in Dentistry to examine the candidates applying for such license. Dr. Palmer was appointed a member of that board when it was organized, and continued in that capacity until he died.

During the whole of his long professional career he continued to be a close student in the different departments of science, particularly in those that could in any way be utilized in the practice of his profession. In the early part of his professional life his investigations were principally carried on in the departments of mechanics and chemistry, but later his investigations were directed to the subject of electricity, in which branch of science he became an enthusiastic student and investigator, which resulted in his promulgating the theory of "vital electricity" and its application to the cure of disease, particularly to those diseases that are treated by the dentist. In this direction he claimed to have made some important discoveries. Like all men who promulgate new ideas, he met with those who opposed his views. But Dr. Palmer was always a careful and patient investigator, and never put forth any theory until he was thoroughly convinced that it was a correct one, and then, having reached his conclusions and promulgated them, he did not seem to care to argue with those who opposed them, but contented himself with stating them clearly and then leaving the matter with the remark, "Well, *time will tell whether I am right or not, and I can afford to wait.*" For, above all things, he disliked a wrangle or a quarrel. In fact, he was always a peacemaker, for nothing seemed to distress him so much as a dispute or quarrel, and when one occurred where he was, either among his friends or during the meeting of a society he was attending, his whole energies and influence would be at once exerted in the cause of peace and good-will. Dr. Palmer was an extensive contributor to the literature of his profession, and his contributions are a valuable part of its scientific accumulations, and they will become more and more valuable when they are better known.

Some years ago Dr. Palmer, in connection with Dr. Flagg, of Philadelphia, for whom he always had a strong friendship, announced their doctrine of the "New Departure" in dental practice, which announcement caused a great sensation in the dental profession of this country. The "New Departure" was received with violent opposition by a large majority of the best practitioners of that time, and it would probably not have been considered at all if it had not been for the great respect that was entertained for the character and attainments of Dr. Palmer. But he was known to be a careful and reliable investigator and a man in whom everybody had faith. Time proved that the new doctrine was generally regarded as true. And Dr. Palmer lived to see it acknowledged as such. So that his motto, "Well, time will tell whether I am right or not," was verified, and time developed the correctness of his theory and his justification in presenting it.

There was combined in his character a large amount of amiability and gentleness, with great firmness of character and an indomitable will, when it became necessary to use it. But in his ordinary intercourse with his fellow-men he was courteous, with a most happy genial manner that made him a most charming and intelligent companion.

Dr. Palmer's father, Avery F. Palmer, was born in Stonington, Conn., where their ancestor, Walter Palmer, from England, first settled in 1629. His great-grandfather, the Rev. Wait Palmer, was pastor of the first Baptist church of North Stonington. Dr. S. B. Palmer was associated with the first Presbyterian church of Syracuse.

Dr. Palmer was a large-hearted man, with a nature overflowing with kindness, good-will, and affection, and to his intimates and friends his personality will ever be a most pleasant memory, for he was one of the truest of men where he gave his friendship or love.

As regards his domestic relations, one who knew him and his family intimately has said of his household, that it was the most harmonious and the happiest one he had ever known.

This could hardly have been otherwise when it is known what a fortunate man he was in obtaining the kind of wife that he did, for in her he had a most congenial and charming companion for the first forty years of his professional life, and it was a fearful blow to him when he lost her. She was a woman blessed with a most cheerful and affectionate disposition, and, like her brother, the Hon. John Savery, of Cato, N. Y., gifted with large mental capacity. She was devoted to her husband, of whom she was very proud, and had entire faith in his ability and the feasibility of his projects. She was always ready to help him in any way that would contribute to the success of his undertakings. And this was done with a cheerful willingness and ready intelligence that made her a most efficient coworker, and she was never so happy as when she was helping him in this way. After the death of his wife, his sister, Mrs. S. C. Brooks, took charge of his household, and he had her affectionate and devoted care and companionship to the end of his life.

His official duties as a dental examiner for registration to practise dentistry brought him in contact with many young men just entering the dental profession. In these and all other young practitioners he was always deeply interested, and to many of them his kindly advice and assistance was always encouraging, and sometimes he was able to direct their career to a successful result, notably in the cases of Dr. John S. Marshall, of Chicago, for it was upon his recommendation that Dr. Allport engaged him as his assistant. Dr. Marshall eventually became Dr. Allport's successor, and thus was enabled to attain an eminent position and a reputation that is world-renowned.

Another instance is Dr. G. Lenox Curtis, of New York, for it was by Dr. Palmer's advice that he qualified himself for the career he is now pursuing as an oral surgeon.

Such evidences of Dr. Palmer's "helping hand" could be multiplied *ad infinitum,* and will make the memory of him dear in many a grateful heart.

His intercourse with his professional brethren was of that character that always commands respect, and those of them who knew him well will remember their acquaintance with him as a bright, green spot in their lives. To his fellow-townsmen his death is an irreparable loss, for he had been a member of their community for over thirty-five years and was known to all the principal people of the town, who respected and honored him as one of their most distinguished citizens. The large attendance at his funeral has shown their appreciation of his worth and their desire to do honor to his memory.

On the day of his funeral his body lay at the First Presbyterian Church, where the services were held, and hundreds assembled there to take a last look at his kindly, familiar face. The Syracuse Dental Society attended in a body, and there were also present over a hundred dentists from different parts of the country. The organizations represented at the funeral services were the National Dental Association, the New York State Dental Society, the Fourth, Fifth, and Seventh District Dental Societies of New York, The Institute of Stomatology of the City of New York, and the Syracuse Dental Society.

The funeral sermon was preached by the Rev. Dr. George B. Spalding, who, in a discourse full of emotional eloquence, declared that the death of Dr. Palmer, while it is a serious loss to the dental profession, was a great calamity to the large circle of devoted friends who were most affectionately attached to him by the sympathetic loveliness of his character.

Dr. Spalding said he had lost a dear friend. "I knew and believed in him as if he had been my father. He was a man without guile, whom you believed in implicitly the moment you looked in his honest, trust-inspiring eyes."

The honorary pall-bearers were Dr. O. J. Gross, of Schenectady; Dr. A. M. Wright, of Troy; Dr. Frank French, of Rochester, who were Dr. Palmer's associates in the Board of State Dental Examiners, and Dr. R. H. Hoffheinz, of Rochester, the president of the New York State Dental Society. The active bearers were Dr. J. H. Dower, Dr. J. E. Cummings, Dr. G. H. Butler, Dr. A. F. Smith, and Dr. C. H. Barnes, all of Syracuse, and Dr. A. D. Wells, of Skaneateles.

The body was taken to Tully for burial, and all the dentists present at the funeral escorted the body from the church to the railroad station.

The case of Dr. Palmer is the only instance in the history of the dental profession where the death of one of its members has created the demonstration of sympathy and sorrow in so large a proportion of a community as large as that of Syracuse. And it illustrates the magnetic character of the man and the influence his splendid qualities had upon the community in which he lived. Hundreds of letters of sympathy and condolence are being received by his friends, many of them paying the highest tribute to his worth and ability. One from Dr. Farrar gives expression to this sentiment: "The great dentist, with his smiling face; his very thoughts could be read by looking in his eyes; he will long live among the thinkers of the profession."

The Rev. George B. Spalding, D.D., his pastor, writes: "His death leaves a great break in the army of dentists. I wish we had more like him."

Dr. James Truman says of him, "He has been to me the embodiment of all that was to be found in dentistry. He has ever been faithful to it, as he has been faithful to the light that made brilliant his inner being and from there reflected to an unbelieving professional world. He was free from dishonesty and hypocrisy. He has a clear record. His death ends a noble life on earth, but does not end the great cycle of activity belonging to an infinite mind."

Thus those who knew him speak of him, all eulogistic of his beautiful character and the splendid gifts he employed so wisely for the benefit of humanity. Requiescat in pace.

## Figures and Tables

**Figure f1:**